# Vesicle-Mediated Transfer of CTX-M β-Lactamase Genes and Proteins Confers Ampicillin Resistance in *Escherichia coli*

**DOI:** 10.3390/ijms262110601

**Published:** 2025-10-31

**Authors:** Nader Kameli

**Affiliations:** 1 Department of Medical Laboratory Technology, College of Nursing and Health Sciences, Jazan University, Jazan 6809, Saudi Arabia; nakameli@jazanu.edu.sa; 2 Health Research Center, Jazan University, Jazan 6809, Saudi Arabia

**Keywords:** *E. coli*, antibiotics resistance, ampicillin, CTX-M, bacterial vesicles

## Abstract

The global rise of antimicrobial resistance represents a critical challenge to public health, with *Escherichia coli* emerging as one of the most significant contributors due to its high adaptability and prevalence of extended-spectrum β-lactamase (ESBL) production. Outer membrane vesicles (OMVs), nanoscale structures released by Gram-negative bacteria, have recently been implicated in the dissemination of resistance determinants and direct antibiotic inactivation. This study investigated the role of OMVs derived from ESBL-producing *E. coli* in mediating resistance to ampicillin. Clinical strains harboring CTX-M-15 resistance genes were cultured under selective pressure, and OMVs were purified via size-exclusion chromatography. Characterization using tunable resistive pulse sensing (TRPS) and cryo-transmission electron microscopy confirmed vesicle integrity, with sizes ranging from 80 to 150 nm. DNA quantification and PCR analysis revealed the presence of CTX-M-15 genes within vesicles, which remained protected from DNase digestion, confirming encapsulation. Functional assays demonstrated β-lactamase activity within OMVs, with proteinase K treatment indicating localization primarily within vesicles rather than on their surface. Importantly, OMVs inactivated ampicillin in a dose-dependent manner, significantly reducing its efficacy against susceptible *E. coli*. Disc diffusion and microtiter plate assays confirmed that β-lactamase-positive OMVs protected susceptible strains from antibiotic killing, promoting bacterial survival and growth. This study uniquely demonstrates that OMVs from CTX-M-15–producing *Escherichia coli* carry both resistance genes and active β-lactamase enzymes, thereby facilitating both genetic dissemination and direct antibiotic inactivation. Targeting OMV biogenesis may represent a novel strategy to combat antimicrobial resistance.

## 1. Introduction

Antibiotic-resistant bacteria represent one of the most significant challenges in contemporary medicine. The inaugural Global Research on Antimicrobial Resistance (GRAM) study, published in 2022, provided the first comprehensive quantification of the global burden of antimicrobial resistance (AMR). The analysis demonstrated that in 2019, AMR was associated with a mortality burden surpassing that of HIV/AIDS and malaria, accounting for an estimated 1.2 million deaths directly attributable to resistant infections and contributing to an additional 4.95 million deaths [1]. Alarmingly, the emergence and dissemination of resistance to broad-spectrum antibiotics—including imipenem, amikacin, quinolones, and piperacillin—have been increasingly reported [2,3,4,5].

*E. coli* is a Gram-negative pathogen of considerable clinical importance, associated with a wide spectrum of diseases. Infections caused by *E. coli* can manifest as diarrhea, vomiting, abdominal pain, and cramping, and in severe cases, may lead to hemolytic–uremic syndrome. In addition to respiratory and wound infections, *E. coli* is the predominant etiological agent of both community-acquired and nosocomial urinary tract infections [6]. The rise in antimicrobial resistance among *E. coli* strains has generated significant global concern [7]. Its highly adaptable genome facilitates the horizontal transfer and clonal dissemination of resistance genes, making it the most prevalent ESBL producer within the *Enterobacteriaceae* family [8]. However, the precise mechanisms of resistance transmission remain incompletely understood.

Understanding the molecular mechanisms underlying resistance, as well as the routes of resistance gene dissemination, is critical for the development of novel antimicrobial agents. Membrane vesicles (MVs)—released by both Gram-negative and Gram-positive bacteria—have emerged as key mediators of bacterial pathogenicity. MV release is generally triggered by environmental stressors, such as antibiotic exposure (chemical) or temperature fluctuations (physical) [9]. In Gram-negative bacteria, outer membrane vesicles (OMVs), ranging in size from 20 to 250 nm [10,11], originate from the outer membrane and retain structural components such as peptidoglycan and lipopolysaccharide (LPS) [12]. OMVs also encapsulate toxins, enzymes, and nucleic acids, including DNA and siRNA [12,13].

OMVs have also been implicated in horizontal gene transfer. *E. coli*-derived OMVs have been shown to carry Shiga toxin genes and transfer them to other Gram-negative species [14]. Furthermore, emerging evidence suggests that OMVs contribute to antibiotic resistance by protecting not only their parent cells but also neighboring bacteria [15,16,17,18].

The present study aims to elucidate the role of OMVs derived from antibiotic-resistant *E. coli* in mediating resistance through direct antibiotic interaction and to determine whether these OMVs can serve as vehicles for resistance gene dissemination.

## 2. Result

### 2.1. Isolation and Quantification of Vesicles

**Figure 1:**
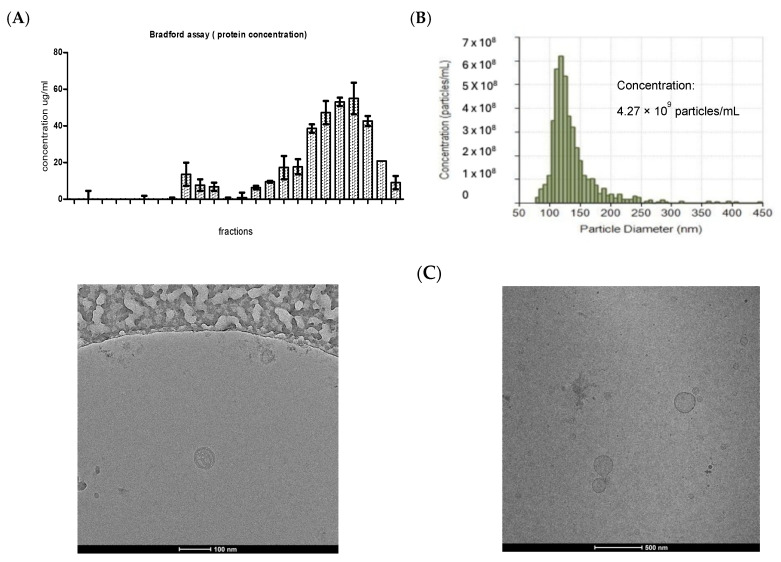
Successful isolation and segregation of particle-containing and protein-containing fractions. Measurement of particle concentrations and evaluation of vesicle-like properties. (**A**) Protein concentrations of all 24 individual SEC fractions (from two OMV isolates), determined by Bradford Assay indicating OMV fractions 8–11. (**B**) images obtained from qNANO showing vesicle concentration and size distribution. (**C**) TEM images, UF_SEC (ultrafiltration followed by SEC) show a successful isolation procedure for OMVs (data represented as mean with SD).

*Escherichia coli* was observed to release outer membrane vesicles (OMVs) at various growth phases, with maximal production during the late logarithmic phase, consistent with prior studies [13,17]. Vesicles isolated by size-exclusion chromatography (SEC) were clearly separated from contaminating bacterial proteins. Bradford assays (Figure 1A) demonstrated that fractions 7–11 contained vesicle-associated proteins at low concentrations, while fractions 17–24 corresponded predominantly to bacterial proteins. Purity of OMV preparations was confirmed by plating filtrates on blood agar, which yielded no bacterial growth. Consistent with previous reports, the highest vesicle concentrations were recovered in fractions 7–12 [19]. These fractions were pooled and quantified by qNano TRPS analysis, which confirmed a particle size distribution of 80–150 nm and a concentration of 4.27 × 10^9^ particles/mL (Figure 1B). OMVs were visualized by cryo-TEM (Figure 1C). Smaller vesicles (<70 nm) observed by cryo-TEM are not fully captured by TRPS due to the lower detection threshold of the nanopore size used; this explains the truncation of the particle distribution.

### 2.2. DNA Quantification

**Figure 2:**
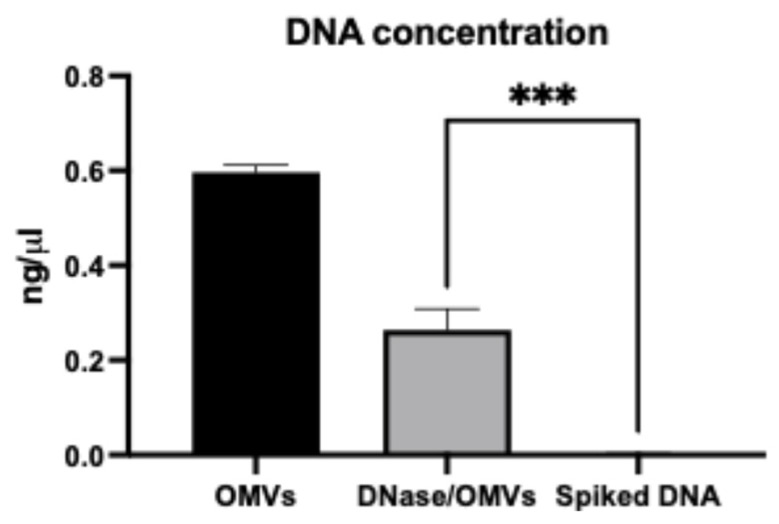
The graph shows the concentration of DNA from different stages of the experiment (OMV lysate and lysate of OMVs prior treated with DNase; spiked DNA followed by DNase treatment). Results indicate that DNA is intact in OMVs. The experiment was conducted twice (data represented as mean with SD) (*** *p*-value < 0.0005).

The presence of vesicle-associated DNA was evaluated using the PicoGreen dsDNA assay. Both untreated and DNase-treated OMVs contained detectable DNA, whereas control samples spiked with exogenous DNA confirmed DNase activity (Figure 2). These findings indicate that DNA was encapsulated within intact vesicles, thereby protected from enzymatic degradation, and prompted further analysis of specific resistance genes.

### 2.3. PCR Analysis

**Figure 3:**
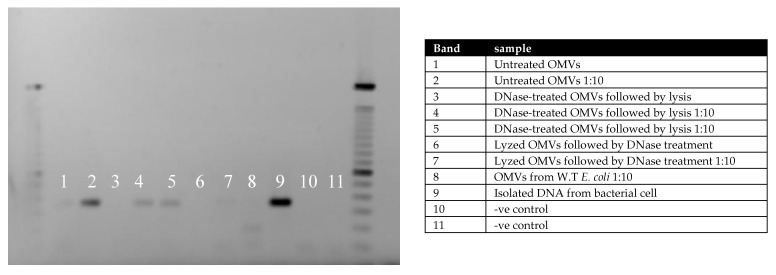
OMVs from resistant bacteria contain resistant genes. MVs were treated with Dnase to get rid of free or membrane-attached DNA followed by lysis as in bands 4 and 5. MVs were also lyzed before being treated with DNase as in bands 6 and 7. Untreated vesicles in bands 1 and 2. The samples were also diluted to avoid possible inhibition of the PCR reaction. Gel-electrophoresis images show the presence.

To assess whether vesicle-derived DNA carried resistance determinants, PCR amplification was performed targeting the CTX-M-15 gene (336 bp). Three sample types were tested: untreated OMVs, DNase-treated OMVs, and DNase-treated wild-type vesicles spiked with CTX-M-15 DNA. Gel electrophoresis revealed the expected amplification product in DNase-treated resistant OMVs (Figure 3), confirming the presence of CTX-M-15 within vesicles.

### 2.4. β-Lactamase Activity

**Figure 4:**
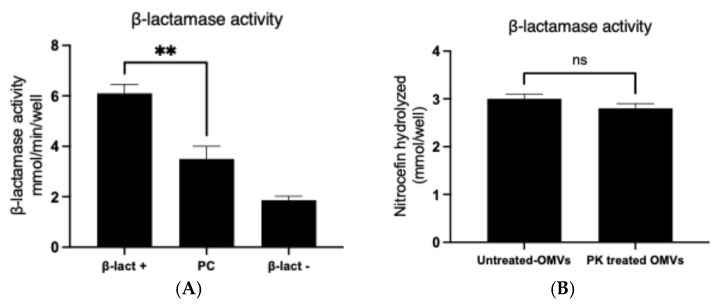
β-lactamase activity of OMVs derived from resistance *E. coli.* The chromogenic substrate nitrocefin was used to determine the activity of β-lactamase. Activity was measured by the ability of an enzyme to hydrolyze the substrate nitrocefin per min per volume added to wells. (**A**) OMVs with β-lactamase positive compared to positive control and vesicles with β-lactamase negative (wild-type). (**B**) The localization of β-lactamase by OMVs treated with proteinase K followed detection of activity; the result showed the presence of enzyme inside the vesicles and pretreatment of OMVs with proteinase K did not significantly alter activity. All measurements were conducted three times (data represented as mean with SD) (** *p*-value < 0.005) (ns: not significant).

Enzymatic activity of OMV-associated β-lactamase was determined using the chromogenic substrate nitrocefin. OMVs derived from β-lactamase-positive strains hydrolyzed nitrocefin at levels exceeding those of the manufacturer-provided positive control (Figure 4A), whereas wild-type vesicles exhibited no activity. Pretreatment of OMVs with proteinase K did not significantly alter activity (Figure 4B), suggesting that β-lactamase was predominantly localized within vesicles rather than attached externally.

### 2.5. Measurement of Ampicillin Efficacy in the Presence of OMVs

**Figure 5:**
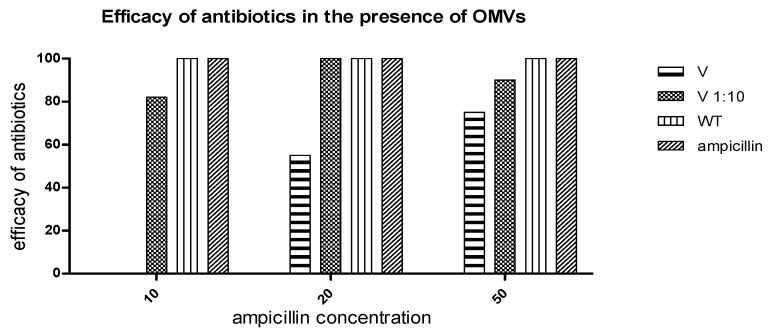
Determination of ampicillin efficacy by Disc diffusion method. Consideration of the Inhibition zone of ampicillin without OMVs as 100% of efficacy. WT which means ampicillin pre-incubated with OMVs derived from wild type β-lactamase negative showed no effect on ampicillin efficacy, while OMVs with β-lactamase positive reduced the efficacy up to 100% with dose-dependence.

**Figure 6:**
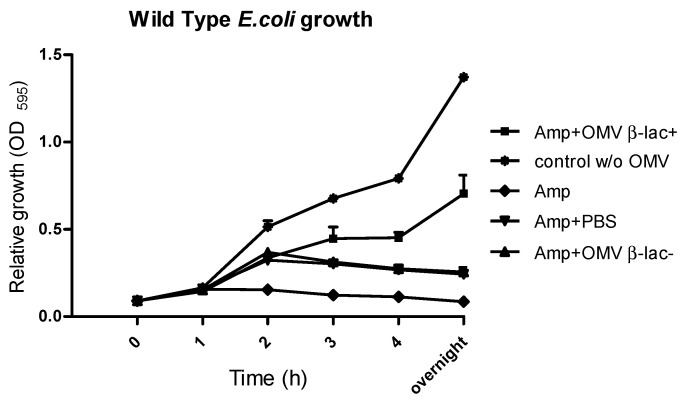
OMVs from β-lactamase positive *E. coli* protect susceptible *E. coli* from ampicillin killing. In total, 10^7^ CFU/mL of susceptible *E. coli* were cultivated with 20 µg/mL of ampicillin that was pre-incubated with either 20 µg/mL β-lactamase positive OMVs or β-lactamase negative OMVs (wild-type) and PBS; in addition to control growth with no OMVs or ampicillin. Absorbance at OD_595_ was expressed as relative growth of bacteria. The result is shown as mean and SEMs of three independent experiments.

The functional impact of OMVs on antibiotic activity was examined using both disc diffusion and microtiter assays. In the disc diffusion assay, Ampicillin pre-incubated with resistant-strain OMVs showed marked loss of efficacy, with no residual antimicrobial effect, indicating that the degradation products lack antibacterial activity. Consistently, microtiter assays demonstrated that β-lactamase-positive OMVs fully abolished ampicillin-induced killing of susceptible *E. coli*, while wild-type vesicles and controls had no effect. OMV doses were quantified as protein per disk (µg) to allow clearer comparison with activity levels, with complete inactivation at 10 µg/mL, ~50% reduction at 20 µg/mL, and ~30% reduction at 50 µg/mL (Figure 5). In contrast, wild-type OMVs had no effect on ampicillin activity. Consistently, microtiter assays demonstrated that β-lactamase-positive OMVs protected susceptible *E. coli* from ampicillin-induced killing, while β-lactamase-negative OMVs and PBS controls did not (Figure 6). Significant differences in bacterial growth were observed as early as 2 h, with protection becoming more pronounced after overnight incubation.

## 3. Discussion

This study demonstrates that *Escherichia coli* outer membrane vesicles (OMVs) can simultaneously (i) shield and transport CTX-M–type β-lactamase genes and (ii) carry catalytically active β-lactamase capable of neutralizing ampicillin, thereby rescuing susceptible bacteria. These findings reinforce—and extend—the emerging view that OMVs are not passive by-products of Gram-negative physiology but are programmable, cargo-selective organelles that reshape antimicrobial exposure at both the cellular and community level [20].

First, our molecular evidence that DNase-resistant vesicles contain CTX-M-15 DNA aligns with work showing vesicle-associated DNA persists extracellularly and is competent for horizontal gene transfer (HGT). DNase protection is consistent with DNA being packaged within the vesicle lumen rather than adsorbed on the outer leaflet, a configuration reported across diverse Gram-negative species and in biofilms, where OMVs can concentrate, protect, and deliver genetic cargo. Such vesicle-mediated HGT has now been described for multiple resistance loci—including *bla*CTX-M, *bla*NDM-1, *bla*OXA-232, and *bla*SHV-12—supporting OMVs as bona fide vectors of resistance dissemination [18,21,22].

Second, our functional data demonstrate that OMVs from ESBL-producing strains reduce ampicillin activity in a dose-dependent fashion and protect otherwise susceptible *E. coli*. This accords with earlier mechanistic studies showing OMV-associated β-lactamases degrade β-lactams in the extracellular milieu, rescuing both clonal siblings and bystanders; indeed, OMVs from β-lactam-resistant *E. coli* have been shown to directly and dose-dependently degrade β-lactam antibiotics and fully rescue susceptible cells. Our observation that proteinase K pretreatment does not abolish activity supports an intraluminal localization of β-lactamase, again consistent with prior reports. Collectively, these data strengthen the concept of “public goods” resistance in which enzymes packaged into OMVs create antibiotic-depleted niches that enable community survival [23,24].

Importantly, our results intersect with mounting evidence that antibiotic exposure modulates OMVs’ biogenesis and cargo. Sub-MIC and cell wall–active agents—including ampicillin—can increase OMV release and alter protein composition, potentially amplifying vesicle-mediated protection precisely when selective pressure is greatest. This positive feedback loop may help explain the rapid collapse of ampicillin efficacy we observed after pre-incubation with β-lactamase-positive OMVs [25].

From a clinical perspective, the CTX-M family remains a dominant driver of community- and hospital-onset ESBL phenotypes. Our data suggest that even in the absence of classic conjugation or transformation, OMVs may expand the ecological reach of CTX-M by simultaneously trafficking the gene and the enzyme, thereby coupling genetic dissemination with immediate phenotypic protection. In polymicrobial niches—urinary tract, gut, and biofilm communities—such dual action could accelerate resistance spread while masking susceptibility during standard testing, with direct implications for β-lactam/β-lactamase inhibitor selection and stewardship [22,24].

These observations motivate several translational strategies. One approach is to target OMV biogenesis, cargo loading, or vesicle–cell interactions. Genetic or chemical interference with envelope stress responses, LPS remodeling, or outer-membrane dynamics can reduce vesiculation, and rational antibiotic design that better penetrates the Gram-negative barrier (or avoids OMVs’ sequestration) is gaining momentum. Complementary tactics include enzyme-directed adjuvants (β-lactamase inhibitors) and agents that disrupt OMVs’ integrity or uptake, thereby preventing extracellular drug hydrolysis and DNA delivery. Finally, given recent advances in OMV detection in complex matrices (e.g., blood), OMVs’ burden and cargo may emerge as dynamic biomarkers for resistance risk and treatment response [26].

Our study has limitations. While PCR and functional assays indicate that CTX-M-15 DNA and β-lactamase reside within OMVs, we did not resolve whether the DNA is plasmid-derived or chromosomal fragments, nor did we directly quantify transformation frequencies into recipients under clinically relevant conditions. Recent reports demonstrate OMV-mediated delivery of intact plasmids and resistance cassettes across genera; extending our model to controlled co-culture and animal infection studies will be essential to establish transfer efficiency, host range, and in vivo impact. Moreover, whereas we focused on ampicillin, OMV-associated β-lactamases (and other enzymes such as carbapenemases) likely differentially impact contemporary β-lactam/β-lactamase inhibitor combinations; systematic profiling across drug classes is warranted [27]. Together, these limitations highlight important avenues for future investigation while reinforcing our conclusion that OMVs act as both carriers of genetic material and extracellular catalysts of antibiotic degradation.

Finally, we noted that nitrocefin permeability across intact OMVs may be limiting. This factor could explain potential kinetic delays observed and represents an important consideration for future research.

In sum, we provide integrated genetic and functional evidence that *E. coli* OMVs encapsulate CTX-M-15 DNA and β-lactamase, jointly mediating ampicillin inactivation and protecting susceptible bacteria. Together with convergent reports across pathogens and settings, these data position OMVs as pivotal—and druggable—determinants of antimicrobial failure. Recognizing OMVs as both couriers of resistance genes and extracellular reactors for antibiotic degradation reframes how we model antimicrobial exposure in vivo and opens new intervention points to curb ESBL spread [22,24].

## 4. Material and Methods

### 4.1. Bacterial Strain and Growth Condition

Two clinical *Escherichia coli* strains (Bu-Uko-99 and J53) harboring plasmids encoding the CTX-M-15 resistance gene were employed in this study. Strains were obtained from the Department of Medical Microbiology, Maastricht University Hospital, and preserved in glycerol stocks at −80 °C. Bacteria were sub-cultured twice on blood agar to ensure optimal growth and verified for antibiotic susceptibility using the VITEK system (bioMérieux, Durham, NC, USA),. Minimum inhibitory concentrations (MICs) were also determined by VITEK. The presence of resistance genes was confirmed by polymerase chain reaction (PCR) using gene-specific primers.

### 4.2. Isolation and Purification of Vesicles

OMVs were harvested during the late logarithmic to early stationary growth phase after 15 h [14,28]. Incubation was conducted in tryptone soya broth (TSB) at 37 °C, 150 rpm, in the presence of 50 µg/mL ampicillin. Following centrifugation at 4000 rpm for 10 min (twice), supernatants were filtered through 0.22 µm syringe filters (Minisart NML syringe filter, Sartorius Stedim Biotech, Göttingen, Germany) and concentrated using 100 kDa Amicon Ultra-15 centrifugal filters (Amicon Ultra 15 mL Centrifugal Filter Unit, Merck Millipore, Billerica, MA, USA) to a final volume of 500 µL. To ensure sterility, filtrates were plated on blood agar. For further purification, samples were subjected to size-exclusion chromatography (SEC) [19] and fractionated into 24 fractions (0.5 mL each). We used a Sepharose CL-2B column (24 mL bed volume) with a flow rate of 0.5 mL/min. Fractions of 0.5 mL were collected, with OMV-rich fractions (7–11) corresponding to V_0_–Vt ranges. Protein concentration was assessed by Bradford assay, and vesicle-enriched fractions (7–11) were pooled and reconcentrated.

### 4.3. Vesicle Quantification and Verification

Vesicle concentration and size distribution were determined by tunable resistive pulse sensing (TRPS) using a qNano Gold instrument (qNano Gold, Izon Science Ltd., Oxford, UK using Izon Control Suite Software v3.2) with an NP150 nanopore. Measurements were calibrated using 200 nm polystyrene beads (SKP200 calibration beads, Izon, Christchurch, New Zealand) provided by the Izon company (1 × 10^9^ particles/mL). For morphological analysis, Cryo-Transmission Electron Microscopy (Cryo-TEM) was used. Three µL of vesicle suspension was applied to glow-discharged holey carbon grids, blotted, and plunge-frozen in liquid ethane using a Vitrobot (FEI, Eindhoven, The Netherlands). Samples were imaged at 200 kV using a Tecnai Arctica cryo-TEM (Thermo Fisher Scientific, Eindhoven, The Netherlands) equipped with a Falcon camera.

### 4.4. DNase Treatment and DNA Quantification

To remove free or membrane-associated DNA, vesicles were treated with DNase I (Fermentas Inc., Lafayette, CO, USA) following a modified Kolling protocol [14]. Briefly, 50 µL vesicle samples were incubated with 3 U DNase I at 37 °C for 10 min, followed by heat inactivation at 70 °C for 10 min. DNase-treated vesicles were lysed by heating at 95 °C for 7–10 min, diluted 1:10, and stored at −80 °C. DNA was quantified at different experimental stages (bacterial lysates, isolated vesicles, DNase-treated vesicles) using the PicoGreen dsDNA assay (Invitrogen, Waltham, MA, USA). Samples were serially diluted (1:10 and 1:100) prior to measurement. DNase-treated OMVs were used exclusively in workflows that subsequently involved full vesicle lysis; therefore, the heating step at 70 °C did not affect intact vesicles or enzymatic assays.

### 4.5. PCR Analysis for Resistant Genes

PCR assays were performed in 25 µL reactions containing Universal SYBR^®^ Green Supermix (Bio-Rad, Hercules, CA, USA) and 10 µM of each primer. Templates included bacterial DNA, vesicle-derived DNA, or DNase-treated vesicle DNA, with wild-type *E. coli* serving as a negative control. Amplification was conducted using a T3000 thermocycler (Biometra GmbH, Göttingen, Germany). PCR products were resolved on 1% agarose gels, stained with ethidium bromide, and visualized under UV light. Primers targeting CTX-M-15 (F: ATGTGCAGYACCAGTAARGTKATGGC; R: ATCACKCGGRTCGCCNGGRAT) yielded a 336 bp amplicon [29]. Cycling conditions included initial denaturation (95 °C, 5 min), followed by 42 cycles of 95 °C (15 s), 58 °C (20 s), and 60 °C (40 s).

### 4.6. Quantification of OMVs β-Lactamase Activity

β-lactamase activity was measured using a chromogenic nitrocefin-based assay kit (Abcam197008, Cambridge, UK). OMVs (25 µg/mL) were incubated in 96-well plates with reaction buffer and nitrocefin substrate. Hydrolysis was monitored at OD490 over 30 min. Enzyme activity was calculated against a standard curve and expressed as nmol nitrocefin hydrolyzed/min/mL. To localize β-lactamase, OMVs were pretreated with proteinase K (100 µg/mL, 1 h, 50 °C), followed by enzyme inactivation using protease inhibitors. All OMV absorbance assays included matched blanks containing OMVs without substrate to correct for light scattering at OD490. Corrected values were used to calculate enzymatic rates.

### 4.7. The Effect of OMVs on Antibiotics

The ability of OMVs to protect susceptible bacteria from ampicillin was assessed by agar diffusion and microtiter assays. Ampicillin (100, 50, 20, 10, and 4 µg/mL) was pre-incubated with OMVs from resistant or susceptible *E. coli* for 1 h at 37 °C [16]. For agar diffusion, 10 µL of each mixture was applied to discs placed on Muller-Hinton agar seeded with wild-type *E. coli* (10^5^ CFU/mL). Zones of inhibition were measured after overnight incubation. For microtiter assays, susceptible bacteria (10^7^ CFU/mL) were cultured with OMV-pretreated ampicillin under 5% CO_2_, and growth was monitored at OD595 over time [16].

### 4.8. Statistical Analyses

All statistical analysis was performed on Graph-Pad Prism 10.5.0 Software (Graph-Pad, San Diego, CA, USA). Differences were considered statistically significant when *p* ≤ 0.05. An unpaired t-test was performed for the statistical analysis of the variance between the means of 2 groups. All additional tests are stated under their corresponding figures.

## Data Availability

This study’s original contributions are included in the article. For further inquiries, please contact the corresponding authors.
